# To avoid carbon degradation in tropical forests, conserve wildlife

**DOI:** 10.1371/journal.pbio.3002262

**Published:** 2023-08-29

**Authors:** Elizabeth L. Bennett, John G. Robinson

**Affiliations:** Wildlife Conservation Society, Bronx, New York, United States of America

## Abstract

Loss of large-bodied wildlife, typically from hunting, degrades the ecological processes in tropical forests that sequester and store carbon. This Perspective argues that carbon-based markets that incentivize wildlife conservation can generate revenues to support necessary forest and hunting management.

## Teaser

Loss of large-bodied wildlife, typically from hunting, degrades the ecological processes in tropical forests that sequester and store carbon. Carbon-based markets that incentivize wildlife conservation can generate revenues to support necessary forest and hunting management.

Many tropical forests have been described as “empty” owing to defaunation (loss of animals), often as a result of unsustainable subsistence or market hunting (Box 1). Such hunting is known to have detrimental effects on target species, broader biodiversity, and the livelihoods and well-being of local communities. Less appreciated is the adverse impact of defaunation on the capacity of tropical forests to sequester and store carbon, which has implications for climate change.

**Box 1. Hunting makes a difference.**

Tropical forests today are empty and quiet. With rare exceptions, they have been emptied of their wildlife by hunting. That is partly for subsistence by forest dwelling people, but hunting for markets has made it unsustainable [1]. Large primates have been impacted by hunting in 32.4% of all sampled forest areas across the Brazilian Amazon, causing losses in population biomass density of 96.97% and 95.80% for spider and woolly monkeys, respectively, in those areas [2]. Across tropical forests globally, large mammal abundances decline by 83% to 90% in hunted compared with unhunted areas, including in protected areas [3]. In Central Africa, between 2002 and 2011, forest elephants lost 62% of their total population and 30% of their geographic range as a result of hunting for the ivory trade, reducing the population to less than 10% of its potential size and occupying less than 25% of its potential range [4].

Many large-bodied mammals and birds that are targeted by hunting are frugivores (fruit eaters) with large gapes that disperse seeds larger than 12 mm; such seeds are characteristic of tree species with high carbon stock capacity [5]. Loss of large frugivores such as primates, hornbills, toucans, and cracids shifts the faunal composition and, over time, the species composition of forests so that wind-dispersed or small-seeded liana and tree species that have lower wood density become more prevalent within the forest stand [6]. Although the picture is complex, with hunting reducing populations of seed predators as well as seed dispersers, the overall effect of over-hunting larger animals is a general reduction in carbon storage capacity. In the Neotropics, for example, defaunation of large primates and tapirs, which disperse seeds from large-seeded trees with higher wood density, is predicted to lead to losses in above-ground tree biomass of 2.5% to 5.8% on average, with some losses as high as 26.5% to 37.8% [3]. In central Thailand, where tree species dependent on seed dispersal by large-bodied frugivores account for nearly one-third of the total carbon biomass, 40% defaunation would result in an estimated carbon reduction of 1% and complete defaunation in a reduction of 2.4% to 3.0% [7].

Hunters often target species whose activities directly affect forest structure. African elephant browsing, for example, tends to reduce the density of tree stems in a forest. The resulting changes in competition for light, water, and space among trees favor the emergence of fewer, larger trees with increased average stem diameter and above ground biomass, and hence, greater carbon stock. At a typical density of 0.5 to 1 elephants per km^2^, this change in forest structure increases the above ground biomass by 26 to 60 t ha^−1^. In central African rainforests, loss of forest elephants results in a 6% to 9% decrease in the above ground biomass [8].

Moreover, defaunation resulting from hunting directly affects total forest carbon storage by removing carbon that is stored in animal bodies. For example, an adult forest elephant holds about 720 kg of carbon (2.64 tons of CO_2_). The 11,000 elephants killed in a single national park in Gabon from 2004 to 2012 [4] would therefore have meant the loss of 7,920 tons of carbon storage, equivalent to 29,040 tons of CO_2_.

Protecting forests is thus an essential part of the global strategy to reduce net carbon emissions by maintaining and growing the carbon sink to absorb the global anthropogenic CO_2_ output. Ecologically intact forests—large, unbroken swaths of forests that are free of significant anthropogenic damage—are of particular importance. High-integrity tropical forests are estimated to remove and store around 3.6 billion tons of CO_2_ per year (net) from the atmosphere [9].

Animals have a vital role in maintaining the integrity of such forests; those forests with their full complement of faunal species, at healthy population densities, sequester and store more carbon than those that have lost components of their fauna. Maintaining intact faunas is therefore a critical component of any strategy to conserve forests to address climate change. Managing hunting is an important part of that and involves a suite of coordinated activities, including working with local hunting communities, effective patrols, monitoring, preventing unsustainable trade of, and reducing demand for, tropical forest wildlife, and appropriate levels of funding to do so.

Restoration of tropical forests by planting seeds and seedlings is emerging as a strategy to reduce net emissions. Gardeners typically work with small-seeded, largely second-growth species. Large-seeded, animal-dispersed tree species are typically underrepresented in seedlings acquired for restoration plantations. Once lost, restoration of animal populations is difficult, especially in the absence of their food sources, and this would constrain the capacity of restored forests to store and sequester carbon [10].

Traditionally, efforts to protect wildlife in tropical forests have been funded by governments and international donors, but allocating funding for wildlife protection has remained a low priority in many parts of the world. Encouraging private investment, and targeting specific outcomes, is an emerging strategy. Sustainability-linked bonds, pioneered through efforts such as rhino bonds, provide a financial incentive for private investors to directly support the conservation of tropical forest fauna. Recent examples include bonds to protect jaguar corridors in Latin America [11] and to rewild land under forest management [12].

Private investments could also be incentivized by linking faunal integrity with forest carbon values. There are already markets that value the carbon sequestration and storage capacity of forests, with REDD+ (Reduced Emissions from Deforestation and forest Degradation) being the most developed. These, mostly voluntary, markets have so far largely focused on the carbon in the forest trees and on reducing emissions by avoiding deforestation and forest degradation. As the loss of large animals degrades the carbon content of the forest, over both the short and long term, there is a market opportunity to incorporate the carbon bonus of an intact fauna.

Such markets will require the provision of an economic value to the contribution that animals make to total forest carbon. An estimate has been made for the effect of African forest elephants on the carbon content of a forest by including the carbon storage value in the body of a single elephant and the contribution of that elephant to a forest’s capacity to sequester and store carbon, both directly, and through its offspring as the elephant population recovers to carrying capacity and expands across its former range over the next 900 years. With these assumptions, the estimated carbon value of a single forest elephant was $1,762,995 [13].

While markets might not be able to capture this long-term asset value, credits could be sold based on the additional carbon in forests that contain healthy populations of large animals. Emission reduction units for forests containing intact faunal assemblages could be “tagged” on that basis, which could potentially increase price, or stimulate additional demand for the credits. The additional funds generated could contribute to the management and conservation of intact forests, including the costs of managing subsistence and market hunting. This approach might provide a partial solution to the problem of how to finance wildlife management in many countries that contain tropical forest. In many of these countries, the primary source of funding has been from international tourism and recreational hunting, the revenue from which can collapse, as occurred during the recent COVID-19 pandemic.

Utilitarian arguments for the conservation of tropical forest wildlife have typically stressed its importance for the health and nutrition of millions of human inhabitants of tropical forests. Sustaining hunted wildlife populations requires the regulation and control of hunting, and/or domestic meat substitution. Institutions and funding to implement the first are generally lacking, while meat substitution, typically by replacing bushmeat with beef, greatly increases the carbon footprint, and the expansion of the livestock production sector in tropical forest countries is a crucial driver of greenhouse gas emissions [14].

Here, we argue that the conservation of well-managed forests with their full faunal communities directly contributes to mitigating global carbon emissions. Hence, explicitly valuing wildlife for its role in the sequestration and storage of carbon in tropical forests, and creating a market for intact faunal assemblages, can potentially generate significant revenues for forest and hunting management. Such a market is one way to pay for the multifaceted programs needed to conserve forests with their full complement of large faunal species, while also ensuring the nutritional health and well-being of local communities in carbon-friendly ways.
